# Facilitators and Barriers Affecting Implementation of Neonatal Palliative Care by Nurses in Mainland China

**DOI:** 10.3389/fped.2022.887711

**Published:** 2022-06-24

**Authors:** Yajing Zhong, Beth Perry Black, Victoria J. Kain, Yang Song

**Affiliations:** ^1^Department of Public Health and Primary Care, Centre for Biomedical Ethics and Law, KU Leuven, Leuven, Belgium; ^2^School of Nursing, University of North Carolina at Chapel Hill, Chapel Hill, NC, United States; ^3^School of Nursing and Midwifery, Griffith University, Nathan, QLD, Australia; ^4^School of Nursing, Guangzhou University of Chinese Medicine, Guangzhou, China

**Keywords:** neonatal palliative care, attitude, facilitators, barriers, neonatal nurses

## Abstract

Neonatal nurses in mainland China encounter various challenges when it comes to delivering palliative care to neonates. The aim of this study was to determine the barriers and facilitators of neonatal nurses' attitudes to palliative care for neonates in mainland China. A simplified Chinese version of the Neonatal Palliative Care Attitude Scale was piloted, administered, and analyzed using survey methods. Nurses in neonatal intensive care units in mainland China regardless of experience in the field were invited to take part in. Over a five-month period in 2019, we surveyed neonatal nurses from 40 hospitals in five provinces of China. The response rate was 92.5% (*N* = 550). This study identified eight facilitators and four barriers to neonatal palliative care implementation. In terms of nurses' attitudes on providing palliative care, younger and older nurses were more positive, whereas middle-aged nurses were less so. Nurses' emotional wellbeing was rarely impacted by neonatal death. They considered neonatal palliative care, particularly pain management, to be just as important as curative treatment. Parents were invited to participate in decision-making by nurses. Nurses reported having access to professional counseling and talking about their concerns with other healthcare professionals. The following barriers to neonatal palliative care were identified in this study that were not observed in the original English version scale research in 2009: a lack of clinicians, time, clinical skills, systematic education, neonatal palliative care experience, and social acceptance. Future research is required to investigate each barrier in order to improve the implementation of neonatal palliative care in mainland China.

## Introduction

In 2019, 6.2 million children under age 5 died, including 2.4 million newborns globally (1). The greatest risk for death for children occurs in the neonatal period (first 28 days after birth) (2). These high rates of mortality mean that healthcare providers across the world encounter ill infants for whom cure is unlikely, therefore providers are increasingly focused on quality of life for both young infants and their families (3, 4).

Accordingly, palliative care for neonates has been an increasingly important element of the lexicon of contemporary neonatal nursing practice in recent years (3, 5), garnering the attention of caregivers and patients' families. Exploration and development have progressed, particularly in the United States, Canada, Australia, and Europe (6, 7).

The World Health Organization supports the concept of end-of-life care in neonatal intensive care units (NICUs) and has encouraged a model of care designed to control pain and achieve the best quality of life for neonates (8), describing palliative care for children as active total care of infants' bodies, minds, and spirits, as well as a means to support the family (9).

Because neonates cannot express their discomfort through language, providing respite for these unwell infants necessitates the provision of expert care (10). Nurses have a pivotal role in neonatal pain management, based on their general knowledge about infants' conditions and specific knowledge about the infants for whom they are providing care (11–13).

Understanding basic principles of neonatal palliative care that maintaining the primacy of palliative care while providing ethical and humane care to support “good death” is not difficult (14), but healthcare providers face a number of difficulties to the actual provision of palliative care (15). Researchers postulated that nurses' attitudes toward palliative care for neonates might be influenced by attitudinal, educational, and institutional issues (16). In 2009, Kain and colleagues reported a composite understanding of barriers and facilitators to palliative care delivery in neonatal nursing, developing, pilot testing and administering an instrument – the Neonatal Palliative Care Attitude Scale – to measure the barriers and facilitators that would be used in subsequent investigations in this field (5).

Hong Kong and Taiwan have drawn on the experience of neonatal palliative care providers in their regions to explore this care in an effort to improve practice (15, 17); however, palliative care for newborns in mainland China is currently almost non-existent, and where implemented, does not receive adequate attention (18–20). In mainland China, neonatal care has been developing over time (21–23). Neonatal nurses undergo specialty training and rotations in different units before becoming neonatal specialist nurses (24). Although neonatal units from primary to tertiary level supported through national healthcare networks (22, 23, 25), the large population of mainland China, the scarcity of nurses and their intense workload, as well as the demands of neonatal palliative care, make implementation difficult (26, 27). Furthermore, little research has been conducted on palliative care practices in mainland China. The concept of neonatal palliative care is still not accepted by the majority of the Chinese population, despite the promulgation of policies/guidelines on the implementation of neonatal palliative care (28, 29). Providers tend to prolong the life span of newborns (22); therefore, in practice, palliative care is still not practiced in most hospitals.

Extant research outside of mainland China has identified the barriers and facilitators that nurses identify in providing neonatal palliative care. Kain in Australia (5), Wright (30), Kyc (31), and Chin (32) in the United States, Cerratti in Italy (33), Chen in Taiwan (34), and Forouzi in Southeast Iran (35) investigated NICU nurses' attitude to neonatal palliative care using the Neonatal Palliative Care Attitude Scale. In addition, Gu explored conditions in mainland China as well (36). However, nurses' attitudes toward neonatal palliative care in China have yet to be investigated in a large scale, therefore their perspectives on this care still needed to be explored further.

The aims of this study are (1) to investigate the attitudes and beliefs of Chinese NICU nurses concerning palliative care for dying neonates; and (2) to identify the barriers and facilitators that may impact nurses' attitudes. We use the term “attitudes” in this study to refer to the measurement of nurses' positive or negative feelings toward caring for dying infants (5).

## Materials and Methods

### Procedures

We jointly submitted the ethical approval application of the pilot study and full study, and our study was deemed exempt by the ethics board of the First Affiliated Hospital of Guangzhou University of Chinese Medicine because it was considered not to -invade the privacy of the participants. This study utilized survey methods to pilot test and administer the Neonatal Palliative Care Attitude Scale. Because the simplified Chinese version of the scale was translated and culturally adapted from the traditional Chinese version (34), we remeasured validity and reliability of the new instrument. The value of KMO was 0.894, which indicated the correlation between variables was strong and factor analysis was valuable to be conducted. We performed a pilot test of the scale to neonatal nurses (*n* = 15) using a cross-sectional survey approach and retested 4 weeks later. Four weeks allowed participants an adequate lapse in time to avoid recall of their answers in the first round. Later, we interviewed five of these participants about the questionnaire's readability because of differences of language expressions between traditional Chinese and simplified Chinese. Assured by the participants that it was comprehendible, we then collected complete data from 550 neonatal nurses using convenience sampling.

### Setting/Participants

We recruited nurses from 40 hospitals (primary, secondary and tertiary hospitals). The size of the NICUs varied, ranging from several to around 50 -cots. Most NICUs did not implement neonatal palliative care yet but have fetal-maternal units, which potentially increased the populations of neonates diagnosed antenatally with congenital malformations. Five hundred fifty participants were registered nurses who practiced in NICUs in mainland China, regardless of experience in the field.

### Research Instrument

The healthcare systems in mainland China and Taiwan differed, and some expressions in traditional Chinese and simplified Chinese are different. In order to be understood by nurses in mainland China, we adjusted the expressions in the simplified Chinese questionnaire. With the approval of the original author Kain (5) and translator Peng (34), we developed and then administered a simplified Chinese version of the Neonatal Palliative Care Attitude Scale, which consisted of 5 subscales and 26 items. The details of the scale development will be published elsewhere.

### Data Collection

Two authors (YZ and YS) contacted nurses in charge in NICUs and got their permission to present our study and distribute questionnaires to neonatal nurses during their breaks. We also administered the simplified Chinese version of the Neonatal Palliative Care Attitude Scale in NICUs through WeChat and a shared URL or QR code. Some nurses in charge helped to introduce our study and distribute questionnaires in the units or via the WeChat group. We sent reminders to participants if they did not complete the questionnaire within 2 days since they got it. We used the WJX platform for questionnaire administration and data collection (https://www.wjx.cn/). We exported and analyzed the data after it was collected to identify factors that may influence attitudes, facilitators, and barriers.

### Data Analysis

Using SPSS, we calculated descriptive statistics to determine frequencies of responses to each item. For interval data, we used independent *t*-tests or ANOVA to make two-group or more than two group comparisons. Statistical significance was set at *p* < 0. 05.

## Results

In the pilot study, we administered the Neonatal Palliative Care Attitude Scale to 15 neonatal nurses from tertiary care hospitals. Cronbach's alpha was 0.867 in the first round and 0.876 in the second round, with a test-retest Pearson r of 0.891. In addition, we asked five nurses to share their perspectives on palliative care in order to see if the questionnaire was clearly understood and to gather any additional feedback on the scale. We proceeded on with the larger study since all of the nurses we interviewed indicated they understood the meaning of the items and had no alternative opinions.

Over a five-month period in 2019, we recruited neonatal nurses from 40 NICUs in five provinces of China (Guangdong, Guizhou, Hebei, Henan, Hunan). We had completed data from 550 (92.5%) of 595 nurses who started the questionnaire; we did not use data from 45 incomplete questionnaires. Table 1 summarizes participants' personal and professional demographic characteristics: 542 (98.5%) were female; ages ranged from 20 to 49 years old (M = 29); years working as NICU nurses ranged from 1 to 28 years (M = 6). Nurses who had cared for dying babies accounted for 68.5% (377); nurses with college degree or above accounted for 56.2% (309); 31.5% (173) nurses have religious beliefs. Of these, 86% (473) nurses worked in tertiary hospitals, 9.8% (54) worked in secondary hospitals, and 4.2% (23) worked in primary, private or other hospitals.

**Table 1:** Personal characteristics (*N* = 550).

**Variables**		***N* (%)**
Gender	Male	8 (1.5)
	Female	542 (98.5)
Age (years)	20–29	308 (56)
	30–39	198 (36)
	40–49	44 (8)
Working years	1–5	306 (55.6)
	6–10	154 (28.0)
	≥10	90 (16.4)
Experience of caring for dying neonates	Yes	377 (68.5)
	No	173 (31.5)
Degree	Technical secondary	21 (3.8)
	Junior college	219 (39.8)
	Undergraduate	306 (55.6)
	Graduate	4 (0.7)
Religion	Yes	174 (31.3)
	No	376 (68.4)
Hospital level	III	473 (86.0)
	II	54 (9.8)
	Private or others	23 (4.2)

### Organization

Kain defined “Organization” as the extent to which the institutional setting in which a neonatal nurse operates presents barriers to and facilitators of palliative care practice (5). Five items were included in the Organization subscale. The overall mean score for this subscale was 3.34 (SD = 0.72), indicating the level of agreement with items listed in this subscale. Most clinicians thought the equipment and environment of healthcare institutions were advantageous for implementation of neonatal palliative care.

### Resources

Kain defined the subscale “Resources” as including physical setting, staffing needs, adjusting the time required to be spent with families, policies and guidelines to support a palliative care model, and whether counseling was available to nurses when a neonate died (5). Five items were included in this subscale and the average score was 2.95 (SD = 0.62), meaning that resources provided by healthcare institutions were inadequate to implement neonatal palliative care.

### Work Experience

This subscale included four items described as work experience for neonatal palliative care (34). The subscale mean was 2.78 (SD = 0.71). The subscale yielded the lowest score among all subscales, demonstrating that many NICU nurses lacked work experience in providing palliative care to dying neonates and their families.

### Beliefs

“Beliefs” were defined as neonatal nurses' thoughts toward palliative care (34). Four items were included and the overall mean score for this subscale was 3.67 (SD=0.65), the highest score among the subscales, indicating many clinicians endorsed implementation of neonatal palliative care and held a positive attitude about this form of care.

### Barriers

The subscale “Barriers” were defined as factors which hindered nurses implementing neonatal palliative care, which included eight items. Item responses in this subscale were reversed. The average score of the subscale was 2.89 (SD = 0.59). Some clinicians tended to implement curative treatment, which might reflect their attitude toward neonatal palliative care. The average score of the subscales were presented in Table 2, item details are described in Table 3.

**Table 2:** Subscales values.

**Subscales**	** $\bar{x}\pm s$ **	**Minimum**	**Maximum**
Organization	3.34 ± 0.72	5	25
Resources	2.95 ± 0.62	5	25
Work Experience	2.78 ± 0.71	5	25
Beliefs	3.67 ± 0.65	3	15
Barriers	2.89 ± 0.59	8	39

*$\bar{x}\pm s$: mean and standard deviation*.

**Table 3:** Responses within the NiPCAS scale (*N* = 550).

**Items**	**Strongly/ Somewhat agreed: *N* (%)**	**Strongly/** **Somewhat disagreed: *N* (%)**	**Unsure: *N* (%)**
**Organization**			
5. The medical staff support palliative care for dying babies in my Unit (median: 3; Q25, Q75: 3, 4)	248 (45.00%)	119 (21.64%)	183 (33.27%)
8. In my Unit, parents are involved in decisions about their dying baby (median: 3; Q25, Q75: 3, 4)	303 (55.09%)	107 (19.45%)	140 (25.45%)
15. In my Unit, when a diagnosis with a likely poor outcome is made, parents are informed of palliative care options (median: 4; Q25, Q75: 3, 4)	282 (51.27%)	133 (24.18%)	135 (24.55%)
16. In my Unit the team expresses its opinions, values and beliefs about providing care to dying babies (median: 4; Q25, Q75: 3, 4)	285 (51.82%)	107 (19.45%)	158 (28.73%)
19. All members of the healthcare team in my Unit agree with and support palliative care when it is implemented for a dying baby (median: 3; Q25, Q75: 3, 4)	237 (43.09%)	113 (20.55%)	200 (36.36%)
**Resources**			
6. The physical environment of my Unit is ideal for providing palliative care to dying babies (median: 3; Q25, Q75: 2, 4)	214 (38.91%)	160 (29.09%)	176 (32.00%)
7. My Unit is adequately staffed for providing the needs of dying babies requiring palliative care and their families (median: 3; Q25, Q75: 2, 4)	194 (35.27%)	208 (37.82%)	148 (26.91%)
13. When a baby dies in my Unit, I have sufficient time to spend with the family (median: 2; Q25, Q75: 2, 3)	123 (22.36%)	322 (58.55%)	105 (19.09%)
14. There are policies/guidelines to assist in the delivery of palliative care in my Unit (median: 3; Q25, Q75: 2, 4)	154 (28.00%)	195 (35.45%)	201 (36.55%)
24. When a baby dies in my Unit, counseling is available if I need it (median: 4; Q25, Q75: 3, 4)	276 (50.18%)	132 (24.00%)	142 (25.82%)
**Work Experience**			
2. I have had experience of providing palliative care to dying babies and their families (median: 3; Q25, Q75: 2, 3.75)	138 (25.09%)	223 (40.55%)	189 (34.36%)
9. My previous experiences of providing palliative care to dying babies have been rewarding (median: 3; Q25, Q75: 2, 4)	170 (30.91%)	171 (31.09%)	209 (38.00%)
11. I am often exposed to death in the neonatal environment (median: 2; Q25, Q75: 2, 4)	147 (26.73%)	347 (63.09%)	56 (10.18%)
18. I have received in-service education that assists me to support and communicate with parents of dying babies (median: 3; Q25, Q75: 2, 4)	173 (31.45%)	215 (39.09%)	162 (29.45%)
**Beliefs**			
1. Palliative care is as important as curative care in the neonatal environment (median: 4; Q25, Q75: 4, 5)	433 (78.73%)	73 (13.27%)	44 (8.00%)
4. There is support for neonatal palliative care in society (median: 3; Q25, Q75: 2, 4)	235 (42.73%)	186 (33.82%)	129 (23.45%)
10. When babies are dying in my Unit, providing pain relief is a priority for me (median: 4; Q25, Q75: 3.25, 4)	412 (74.91%)	78 (14.18%)	51 (9.27%)
12. Palliative care is necessary in neonatal nursing education (median: 4; Q25, Q75: 3, 4)	405 (73.64%)	67 (12.18%)	78 (14.18%)
**Barriers**			
3. I feel a sense of personal failure when a baby dies (median: 4; Q25, Q75: 2, 4)	180 (32.73%)	330 (60.00%)	40 (7.27%)
17. Caring for dying babies is traumatic for me (median: 4; Q25, Q75: 2, 4)	154 (28.00%)	310 (56.36%)	86 (15.64%)
20. In my Unit, the staff go beyond what they feel comfortable with in using technological life support (median: 3; Q25, Q75: 2, 4)	174 (31.64%)	243 (44.18%)	133 (24.18%)
21. In my Unit, staff are asked by parents to continue life-extending care beyond what they feel is right (median: 4; Q25, Q75: 2, 4)	147 (26.73%)	283 (51.45%)	120 (21.82%)
22. There is a belief in society that babies should not die, under any circumstances (median: 3; Q25, Q75: 2, 4)	188 (34.18%)	217 (39.45%)	145 (26.36%)
23. Palliative care is against the values of neonatal nursing (median: 2; Q25, Q75: 2, 3)	332 (60.36%)	87 (15.82%)	131 (23.82%)
25. There is a belief in society that babies should not die, under any circumstances (median: 2; Q25, Q75: 2, 4)	295 (53.64%)	160 (29.09%)	95 (17.27%)
26. Curative care is more important than palliative care in the neonatal intensive care environment (median: 4; Q25, Q75: 2, 4)	143 (26.00%)	316 (57.45%)	91 (16.55%)

### Factors May Influence Attitudes

We grouped participants into three age groups based on their decade of life: 20–29 years old (*n* = 308, 56%), 30–39 years old (*n* = 198, 36%), and 40–49 years old (*n* = 44, 8%) with average age of 29.3 years. We used one-way ANOVA to determine any differences in scale scores by age and found that scores differed significantly across ages (F = 6.466, P = 0.002). Differences in attitude scores were statistically significant between nurses aged 20–29 and nurses aged 30–39 years old (*P* = 0.001), between nurses in their 30s and those aged 40–49 years old (*P* = 0.035). Nurses in their 40 s (M = 3.15, SD = 0.38) and nurses in their 20 s (M = 3.13, SD = 0.39) had higher scores; nurses in their 30 s had slightly lower scores (M = 3.01, SD = 0.37).

We created three groups of nurses by years of work experience: 1–5 years (*n* = 306, 55.6%), 6–10 years (*n* = 154, 28%), and >10 years (*n* = 90, 16.4%). Difference in scores across the three groups were statistically significant (F = 4.130, *P* = 0.017): nurses who had worked for 1–5 years and those who had worked for 6–10 years were significantly different (*P* = 0.004). Nurses with >10 years' work experience (M = 3.12, SD = 0.38) and with 1–5 years' work experience had a higher (more positive) attitude scores (M = 3.10, SD = 0.36). Nurses who had worked for 6–10 years had somewhat lower scores (M = 3.02, SD = 0.41).

To summarize, we found that nurses aged 20–29, and who had worked for 1–5 years had higher attitude scores and held slightly more positive attitudes toward palliative care. Most nurses ages 40–49, and who had worked >10 years in neonatal units also had high scores, while nurses ages 30–39, with 6–10 years of work experience had lower attitude scores, and thus might hold a negative attitude toward neonatal palliative care (Table 4).

**Table 4:** Age and work years crosstab.

**Age/Work years**	**1–5 years**	**6–10 years**	**>10 years**
20**–**29 years	248	59	1
30**–**39 years	51	90	57
40**–**49 years	7	5	32

### Facilitators and Barriers

Facilitators derived from Organization, Resources, Barriers and Beliefs subscales. In the Organization subscale, parents were welcome to participate in neonates' healthcare decisions (median: 4; Q25, Q75: 3, 4); clinicians might introduce the concept of neonatal palliative care to families when neonates had severe conditions (median: 4; Q25, Q75: 3, 4); clinicians could discuss their opinions, values and beliefs with each other (median: 4; Q25, Q75: 3, 4). In Resources subscale, nurses could get required consultation included psychological comfort (median: 4; Q25, Q75: 3, 4). In Beliefs subscale, many nurses believed neonatal palliative care was as important as curative treatment (median: 4; Q25, Q75: 4, 5). While both hold great importance (median: 4; Q25, Q75: 2, 4), nurses regarded relieving pain as primary task when a neonate is seriously or terminally ill (median: 4; Q25, Q75: 3.25, 4). Nurses endorsed the inclusion of palliative care in nursing education as well as hospital training to improve nurses' understanding about this model of care (median: 4; Q25, Q75: 3, 4). In the Barriers subscale, few nurses experienced negative feelings when caring dying neonates (median: 4; Q25, Q75: 2, 4). They appreciated the importance of neonatal palliative care and wanted to be able to mention it to families (median: 4; Q25, Q75: 2, 4). Furthermore, neonatal nurses were rarely frustrated by the deaths of neonates because they encountered this frequently (median: 4; Q25, Q75: 2, 4).

Barriers were also identified in the Resources, Beliefs and Work Experience subscales. In the Work Experience subscale, nurses had rarely encountered neonatal palliative care in practice so they have had few opportunities to consider if they had difficulty providing it (median: 2; Q25, Q75: 2, 4). In the Resources subscale, nurses noted that the restrictions on the care they can provide, especially psychological care to families, because of their heavy workload (median: 2; Q25, Q75: 2, 3). In the Barriers subscale, neonatal nurses disagreed with palliative care values that affected their attitudes (median: 4; Q25, Q75: 2, 3). With minimal public awareness of neonatal palliative care, families insisted that healthcare providers resuscitate newborns regardless of the infant's condition (median: 4; Q25, Q75: 2, 4) (Table 3).

## Discussion

### Nurses' Attitudes

In mainland China, three factors appear to positively affect use of neonatal palliative care: (1) support of neonatal palliative care in health care institutions; (2) social support for families to be involved in healthcare decisions; and (3) opportunities to express one's opinions, values and beliefs. Nurses endorsed the significance of neonatal palliative care and considered pain relief as a primary tenet of palliative care, and support inclusion of palliative care in neonatal education at universities and specialized training in hospitals.

Nurses' workloads were too onerous to support this type of care due to limited resources or clinicians in healthcare organizations to create suitable neonatal palliative care environments. There were no standards or protocols for nurses to follow when adopting neonatal palliative care, and the psychological counseling required by healthcare providers was not provided. Many healthcare providers have never previously delivered neonatal palliative care or were dissatisfied with their palliative care experience. Nurses trained in neonatal palliative care would likely face the death of their neonatal patients on a regular basis, which could be painful or frustrating for them; however, many nurses did not believe they were capable of providing expert neonatal palliative care to babies and families because they had not yet received structured palliative care education. It was thought that parents may hope that nurses will attempt to extend an infant's life despite the baby's incurable condition, yet curative treatment may cause the newborn greater pain. Nurses administer curative measures in accordance with the intentions of the parents. Importantly, some nurses in our study concluded that curative treatments were more important than palliative care because palliative care violated neonatal care values.

### Factors Affecting Nurses' Attitudes

Nurses' attitude toward palliative care differed statistically across nurses' ages and years of work, although the difference in scores was very small. Some studies indicated older nurses with rich caring experiences had more positive attitudes toward supportive care of patients approaching the end of life (37, 38). With experience afforded by their years of nursing, older nurses may simply have cared for more persons who are terminally ill than younger nurses, and hence may provide higher quality of care (39). Clinical experience is a factor enhancing attitudes toward care of dying persons among nurses and is consistent with the view that older age and more experience may have a positive effect on nurses' attitudes toward care of dying persons. Conversely, other researchers have concluded that nurses' age was irrelevant regarding their attitudes toward end-of-life care (40). In our study, younger nurses with less work experience and older nurses with rich care experience have higher scores. Our research does not explain this; we speculate that younger nurses may have received palliative care education during training but lack extensive experience providing this care; and older nurses may feel more comfortable with palliative and end-of-life care due to their extensive work experience. Nurses in their 30 s may have lower ratings due to a lack of palliative care education and long-term work experience, which can add to their comfort level when delivering palliative care to newborns.

Nurses' caring knowledge is critical to children's development. Neonatal care education in mainland China focuses more on prolonging and maintaining neonates' life (24, 41–44). Caring or nursing education programs consider and progress in the nurturing, feeding, treatment, and reassurance of newborns (24, 42–44). Nursing students or nurses have received education or specialized training in neonatal curative treatments prior to or during their careers (24). However, very few nursing school or hospital curricula include palliative care education or training programs (29), which may explain nurses' lack of knowledge about neonatal palliative care.

### Facilitators and Barriers

We identified eight facilitators and four barriers to implementation of neonatal palliative care among the 550 NICU nurses in mainland China who participated in our study. Facilitators included: nurses admitted the importance of neonatal palliative care, pain relief, palliative care education; neonatal palliative care introduction to families; parents involvement in decision-making; avoidance of negative emotions to neonatal death; professional psychological consultation to nurses and opportunities to express opinions, values or beliefs. Barriers were: limited neonatal palliative care requirement; insufficient nurses or time; and low acceptance of neonatal palliative care by nurses or the society.

### Comparison

In comparing our findings with those of Gu and colleagues from their study conducted in mainland China (36), we found both commonalities and differences. Gu noted the relationship between attitude scores and personal characteristics were not statistically significant; however, in our study, younger nurses aged 20 to 29 years with less work experience and older nurses > 40 years old with rich care experience scored higher. Facilitators discussed in the study of Gu et al. (36), including support from clinicians, emphasis on pain management and neonatal palliative care education, were also involved in our study. Moreover, we found extra facilitators: neonatal palliative care communication with families; parental involvement in end-of-life decision-making process; opportunities for nurses to express perceptions and to ask for psychological support; as well as nurses' ways to avoid passive emotions.

In addition, we found different barriers from those discussed by Gu et al. (36). The common barrier with our study is that some nurses agreed curative treatment is more important than palliative care and violated neonate care values. Other barriers were found in the study of Gu et al. (36) were clinicians' discomfort with the use of technological life support, parental requirements for curative treatment and clinicians' negative feelings when cared a dying baby or when a baby died. We assume these significant differences came from the composition of Gu's sample, which involved neonatologists who expressed stronger attitudes toward implementing neonatal palliative care than nurses (36).

Compare with the study of Chen et al. (34) conducted in Taiwan, some similarities and differences exist as well. First, regarding age and years of work, Chen reported that nurses over 30 years old with longer work experience might hold more positive attitudes about neonatal palliative care. Our study also supported this finding in the participants with higher scores were > 40 years old with extensive care experience; however, nurses aged 20 to 29 years old had higher scores than nurses in their 30 s. Second, both studies found the lack of supportive policies or guidelines to guide nurses to implement neonatal palliative care. These two studies, however, had differences. Chen indicated nurses' attitudes to neonatal palliative care were highly affected by their attitudes about death and willingness to deliver this care; our study, however, found nurses' views on death might not influence their attitudes toward palliative care. In Taiwan, cultural issues and religion were crucial to nurses' willingness to implement palliative care, while religion was not a significant factor among nurses in mainland China. Further study is required to explore these factors.

Even though medical systems in Taiwan and mainland China differ, these two studies have similar barriers to the implementation of neonatal palliative care. Nurses in both studies reported having insufficient palliative care education, so their knowledge may be insufficient to implement care. They also noted having insufficient numbers of healthcare providers to implement neonatal palliative care, including providing comfort to families. Both Taiwan and our study noted lack of supportive policies or guidelines and their lack of professional care experience in providing neonatal palliative care. In addition, parents often demand continuation of care for critically ill neonates regardless of likelihood of success. Some barriers, however, were in sharp contrast. Compared to nurses in our study, Taiwanese nurses noted they were discouraged from discussing opinions, values or beliefs about neonatal palliative care; they could not get counseling when they encountered problems; the imperative to use technologies might result in overtreatment and increase ethical and moral debates, which made most participating nurses felt uncomfortable with the implementation of neonatal palliative care; and nurses' views on death might affect their willingness to deliver palliative care.

The study of Kain et al. (5) in Australia revealed two facilitators to neonatal palliative care that nurses in our study did not mention: (1) professional guidelines; and (2) parents' support. Additional barriers noted by nurses in the Australian study were: (1) technological imperatives; and (2) lack of ideal physical environment. Among studies on neonatal palliative care completed in the United States (30–32), participants identified facilitators not mentioned in our study: (1) parents were informed of neonatal palliative care options; (2) nurses in the United States thought their hospitals have sufficient numbers of experienced clinicians to provide palliative care to infants and parents; and (3) nurses had the support of policies/guidelines to implement neonatal palliative care successfully. Nurses in the United States noted these barriers: (1) inability to express opinions, values or beliefs; (2) no ideal physical environment to implement neonatal palliative care; (3) technological imperatives; (4) lack of in-service education. In a study conducted in Italy, investigators reported facilitators not mentioned in our study (33): (1) endorsement of religious values; and (2) multidisciplinary team support. Italian nurses identified these barriers: (1) lack of knowledge of neonatal palliative care; (2) lack of support of team members; (3) absence of concrete policies/guidelines; (4) poor implementation environment; (5) difficulties in technology implementation; and (6) negative emotions. Because the tenets of palliative care were developed in western countries such as Australia, the United States and Italy, the social acceptance and support in these countries by both healthcare providers and parents facilitate the practice of neonatal palliative care. Because the field of palliative care is new in mainland China, neonatal palliative care has yet to be fully developed, practiced and accepted.

### Limitations

Because the subscales used in the research were different from the traditional Chinese version of the Neonatal Palliative Care Attitude Scale and from the original scale, some items were not strongly correlated across versions. Improving the simplified Chinese version of the scale would be a useful direction for future research. Also, we note that the study conducted in Taiwan to which we compared our findings were done several years ago. This means that nurses in Taiwan might have attitudes about neonatal palliative care that diverge even more from nurses in mainland China, whose attitudes we measured more recently.

## Conclusions

Despite the fact that the field of neonatal palliative care continues to be developed, neonatal palliative care is rarely mentioned in mainland China. Traditional cultural beliefs and practices pose challenges to the prevalence of palliative care in mainland China, where the view of life and death is that discussing death is a symbol of misfortune and fear. According to the traditional medical Taoist view, as long as a patient still has a trace of life, clinicians should use all possible measures to treat the patient until death. This could be a substantial sociocultural barrier to neonatal palliative care use since it represents strongly held beliefs that are difficult to modify.

In this study, we identified facilitators and barriers to neonatal palliative care implementation and found that neonatal nurses in mainland China have a positive attitude toward the implementation of palliative care for ill newborns and believe that neonatal palliative care is of great significance. A major barrier to neonatal palliative care implementation was inadequate nurse staffing, leading to a heavy workload, and thus minimizing the amount of time they need to practice neonatal palliative care adequately. Further study is needed to explore each barrier to neonatal palliative care, with the goal of improving neonatal nurses' attitudes and beliefs toward palliative care and its implementation in mainland China.

## Data Availability Statement

The original contributions presented in the study are included in the article/Supplementary Material, further inquiries can be directed to the corresponding authors.

## Ethics Statement

The study involving human participant was reviewed and was deemed exempt by the Ethics Board of the First Affiliated Hospital of Guangzhou University of Chinese Medicine. The patients/participants provided their written informed consent to participate in this study.

## Author Contributions

YZ, BB, VK, and YS made a substantial contribution to the concept or design of the work, or acquisition, analysis or interpretation of data, drafted the article or revised it critically for important intellectual content, approved the version to be published, and have participated sufficiently in the work to take public responsibility for appropriate portions of the content.

## Funding

The study was supported by a grant from Guangdong Academic of Social Science (Grant No. GD20CGL06).

## Conflict of Interest

The authors declare that the research was conducted in the absence of any commercial or financial relationships that could be construed as a potential conflict of interest.

## Publisher's Note

All claims expressed in this article are solely those of the authors and do not necessarily represent those of their affiliated organizations, or those of the publisher, the editors and the reviewers. Any product that may be evaluated in this article, or claim that may be made by its manufacturer, is not guaranteed or endorsed by the publisher.
