# Supplementary material for: Facilitators and Barriers Affecting Implementation of Neonatal Palliative Care by Nurses in Mainland China
Source: Front Pediatr. 2022 Jun 24;10:887711. doi: 10.3389/fped.2022.887711 (PMC9263274; doi:10.3389/fped.2022.887711)
Supplement: Supplementary file 1 [file Table_1.DOCX]

新生儿姑息护理态度调查表

The Neonatal Palliative Care Attitude Scale (NiPCAS)

非常感谢您在百忙之中接受我们的问卷调查。此问卷的设计是用来探讨国内护理人员有关濒临死亡之新生儿安宁疗护（姑息护理）的态度及信念。我们期望您依据“非常同意”，“比较同意”，“不太同意”，“完全不同意”，“不确定”的程度回答此问卷的问题。问卷采取无记名方式，且仅供研究分析，不会做其它用途，敬请放心作答！

Thank you very much for taking this questionnaire out of your busy schedule. This questionnaire was designed to explore the attitudes and beliefs of domestic clinicians regarding neonatal palliative care. We expect you to rate your attitudes and beliefs on the basis of "Strongly Agree", "Somewhat Agree", " Somewhat Disagree ", " Strongly Disagree", and "Unsure". The questionnaire is anonymous and is intended for research and analysis purposes only, and will not be used for other purposes.

非常同意 = Strongly Agree; 比较同意 = Somewhat Agree; 不太同意 = Somewhat Disagree; 完全不同意 = Strongly Disagree; 不确定 = Unsure

1. 在新生儿科内，姑息护理措施跟治疗性护理一样重要

Palliative care is as important as curative care in the neonatal environment

1. 非常同意 B. 比较同意 C. 不太同意 D. 完全不同意 E. 不确定
2. 我曾有过提供姑息护理给濒死婴儿及他们家人的经验

I have had experience of providing palliative care to dying babies and their families

1. 非常同意 B. 比较同意 C. 不太同意 D. 完全不同意 E. 不确定
2. 婴儿死亡时，我感觉到个人的挫败

I feel a sense of personal failure when a baby dies

1. 非常同意 B. 比较同意 C. 不太同意 D. 完全不同意 E. 不确定
2. 我相信社会上的大多数人都同意新生儿姑息护理的观点

There is support for neonatal palliative care in society

1. 非常同意 B. 比较同意 C. 不太同意 D. 完全不同意 E. 不确定
2. 我的工作单位内，医护人员支持提供濒死婴儿姑息护理

The medical staff support palliative care for dying babies in my Unit

1. 非常同意 B. 比较同意 C. 不太同意 D. 完全不同意 E. 不确定
2. 我工作单位的设备可提供濒死婴儿理想的姑息护理环境

The physical environment of my Unit is ideal for providing palliative care to dying babies

1. 非常同意 B. 比较同意 C. 不太同意 D. 完全不同意 E. 不确定
2. 我工作单位内有充足的工作人员，能提供濒死婴儿及其家属所需的姑息护理

My Unit is adequately staffed for providing the needs of dying babies requiring palliative care and their families

1. 非常同意 B. 比较同意 C. 不太同意 D. 完全不同意 E. 不确定
2. 我工作单位内，双亲能共同参与关于濒死婴儿的决定

In my Unit, parents are involved in decisions about their dying baby

1. 非常同意 B. 比较同意 C. 不太同意 D. 完全不同意 E. 不确定
2. 我对过去提供姑息护理给濒死婴儿的经验感到满意

My previous experiences of providing palliative care to dying babies have been rewarding

1. 非常同意 B. 比较同意 C. 不太同意 D. 完全不同意 E. 不确定
2. 我认为面对濒死婴儿时，缓解疼痛是我的首要任务

When babies are dying in my Unit, providing pain relief is a priority for me

1. 非常同意 B. 比较同意 C. 不太同意 D. 完全不同意 E. 不确定
2. 在新生儿科里，我经常面对死亡

I am often exposed to death in the neonatal environment

1. 非常同意 B. 比较同意 C. 不太同意 D. 完全不同意 E. 不确定
2. 新生儿医护教育有必要包括姑息护理

Palliative care is necessary in neonatal nursing education

1. 非常同意 B. 比较同意 C. 不太同意 D. 完全不同意 E. 不确定
2. 我工作单位里若婴儿死亡，我有充足的时间陪伴他的家属在一起

When a baby dies in my Unit, I have sufficient time to spend with the family

1. 非常同意 B. 比较同意 C. 不太同意 D. 完全不同意 E. 不确定
2. 我工作单位内有一套实施姑息护理的政策或工作指引

There are policies/guidelines to assist in the delivery of palliative care in my Unit

1. 非常同意 B. 比较同意 C. 不太同意 D. 完全不同意 E. 不确定
2. 我工作单位内，若婴儿经诊断后可能具有极差的预后，医护人员会告知双亲姑息护理的概念

In my Unit, when a diagnosis with a likely poor outcome is made, parents are informed of palliative care options

1. 非常同意 B. 比较同意 C. 不太同意 D. 完全不同意 E. 不确定
2. 我工作单位内，医疗团队同仁对提供濒死婴儿姑息护理一事，表达各自的意见、价值观或信念

In my Unit the team expresses its opinions, values and beliefs about providing care to dying babies

1. 非常同意 B. 比较同意 C. 不太同意 D. 完全不同意 E. 不确定
2. 照护濒死婴儿给我带来痛苦的感受

Caring for dying babies is traumatic for me

1. 非常同意 B. 比较同意 C. 不太同意 D. 完全不同意 E. 不确定
2. 我接受过相关的在职教育训练，足以让我提供支持给濒死婴儿的双亲，也能与他们沟通无障碍

I have received in-service education that assists me to support and communicate with parents of dying babies

1. 非常同意 B. 比较同意 C. 不太同意 D. 完全不同意 E. 不确定
2. 应用姑息护理于濒死婴儿时，我工作单位内的健康照顾人员均同意且支持这项做法

All members of the healthcare team in my Unit agree with and support palliative care when it is implemented for a dying baby

1. 非常同意 B. 比较同意 C. 不太同意 D. 完全不同意 E. 不确定
2. 在工作单位内，工作人员即使觉得不妥当，仍采取仪器维持婴儿的生命

In my Unit, the staff go beyond what they feel comfortable with in using technological life support

1. 非常同意 B. 比较同意 C. 不太同意 D. 完全不同意 E. 不确定
2. 我工作单位内，即使工作人员不认同生命延续照护，但在父母要求下，仍勉为其难的进行

In my Unit, staff are asked by parents to continue life-extending care beyond what they feel is right

1. 非常同意 B. 比较同意 C. 不太同意 D. 完全不同意 E. 不确定
2. 我对于死亡的态度常影响我提供姑息护理的意愿

My personal attitudes about death affects my willingness to deliver palliative care

1. 非常同意 B. 比较同意 C. 不太同意 D. 完全不同意 E. 不确定
2. 姑息护理为违反新生儿照护的价值观

Palliative care is against the values of neonatal nursing

1. 非常同意 B. 比较同意 C. 不太同意 D. 完全不同意 E. 不确定
2. 我工作单位内如有婴儿死亡，若我有需要，可获得（心理）咨询

When a baby dies in my Unit, counselling is available if I need it

1. 非常同意 B. 比较同意 C. 不太同意 D. 完全不同意 E. 不确定
2. 有一种信念，认为无论发生什么状况，婴儿都不该死亡

There is a belief in society that babies should not die, under any circumstances

1. 非常同意 B. 比较同意 C. 不太同意 D. 完全不同意 E. 不确定
2. 新生儿科内，治疗性照护比姑息护理重要

Curative care is more important than palliative care in the neonatal intensive care environment

1. 非常同意 B. 比较同意 C. 不太同意 D. 完全不同意 E. 不确定
