# Supplementary material for: Facilitators and Barriers Affecting Implementation of Neonatal Palliative Care by Nurses in Mainland China
Source: Front Pediatr. 2022 Jun 24;10:887711. doi: 10.3389/fped.2022.887711 (PMC9263274; doi:10.3389/fped.2022.887711)
Supplement: Supplementary file 2 [file Table_2.DOCX]

**Translation and cultural adaptation**

**Terminology adaptation:**

*For the whole scale*

1. “palliative care”: adapt “安宁疗护” as “姑息护理”.

2. “somewhat agreed”: adapt “某些同意” as “比较同意”.

3.: “somewhat disagree”: adapt “某些不同意” as “不太同意”.

4. “neonatal environment”, “neonatal intensive care environment”: adapt “新生儿加护病房” as “新生儿科”.

5. :health care team: adapt “健康照顾人员” as “医护人员”.

*For individual items*

6. Item: I feel a sense of personal failure when a baby dies.

Chinese: 婴儿死亡时，我感觉到个人的失败.

Adapt “失败” as “挫败”.

7. Item: There is a belief in society that babies should not die, under any circumstances.

Chinese: 社会上有股信念，认为无论发生什么状况，婴儿都不该死去.

Adapt “有股信念” as “有一种信念”, adapt “死去” as “死亡”.

8. Item: Curative care is more important than palliative care in the neonatal intensive care environment.

Chinese: 新生儿加护病房内，治愈性照护比姑息护理重要.

Adapt “新生儿加护病房“ as “新生儿科”, “治愈性照护” as “治疗性照护”.

9. Item: In my unit the team expresses its opinions, values and beliefs about providing care to dying babies

Chinese: 我工作单位内，医疗团队同仁对提供濒死婴儿姑息护理一事，表达各自的意见、价值观及信念.

Adapt “及” as “或”.

10. Item: There is support for neonatal palliative care in society.

Chinese: 社会上对新生儿安宁疗护提供充足的支持.

Adapt as 我相信社会上的大多数人都同意新生儿姑息护理的观点.

11. Item: My unit is adequately staffed for providing the needs of dying babies requiring palliative care and their families.

Chinese: 我工作单位内有充足的工作人员，能提供濒死婴儿所需的安宁疗护，以及他们家属之所需.

Adapt as 我工作单位内有充足的工作人员，能提供濒死婴儿及其家属所需的姑息护理.

12. Item: Caring for dying babies is traumatic for me.

Chinese: 对我而言，照护濒死婴儿让我深受创伤.

Adapt as 照护濒死婴儿给我带来痛苦的感受.

**Deletion**

13. Item: When a baby dies in my unit, I have sufficient time to spend with the family.

Traditional Chinese: 我工作单位内若婴儿死亡，我有充足的时间陪伴他的家属在一起，并提供必要的协助.

Delete “并提供必要的协助”.

14. Item: There are policies/guidelines to assist in the delivery of palliative care in my unit.

Chinese: 我工作单位内有一套实施姑息护理的政策或/工作指引.

Delete: “/”.
